# Reports from the Laboratory of the Royal College of Physicians, Edinburgh

**Published:** 1893-06

**Authors:** 


*Reports from the Laboratory of the Royal College of Physicians, Edinburgh.* Vol. IV. Pp. 254. Edinburgh: YOUNG J. PENTLAND. 1892.

This volume quite attains the usual high standard of these *Reports*. It comprises a number of papers of great interest and of high scientific value.

Amongst the longer contributions are two on the Anatomy of the Female Pelvis, by Dr. Webster. Drs. Berry Hart and Lovell Gulland contribute a paper on the Structure of the Human Placenta, illustrated by many beautiful plates of the microscopic appearances, in which they adduce evidence to show that, whilst the serotina is forming, the ovum becomes embedded in it, the serotina reflexa being merely the superficial portion of it, and the serotina proper the deep portion of it. They also believe that the ovum can only graft itself on connective tissue, that there is a special area for safe grafting, which is denuded of epithelium during menstruation, and that it can graft itself in the Fallopian tube only when the epithelium has been removed by disease.

Dr. Noël Paton publishes observations on the valves of the mammalian heart, on the composition and uses of the bile in man (supplementary to a paper in a former volume of these *Reports*), and a full account of a remarkable crystalline globulin which was found in the urine of a patient of Dr. Byrom Bramwell's. The detailed clinical history of this case has been published in the latter's *Atlas of Clinical Medicine.*

Dr. Ralph Stockman contributes a pharmacological study of Urechites suberecta, a drug belonging to the digitalis group, but unlikely to be of therapeutic value on account of its possessing in marked degree the cumulative properties of this group. He has also a paper on thebaïne, narcotine, and certain of their derivatives.

Dr. Alexander Miles, in a thesis which gained the Syme Surgical Fellowship, discusses the mechanism of cranial injuries. He has treated the subject in a masterly way, his conclusions are drawn both from experimental and clinical data, and the paper is one which deserves careful study. It is accompanied by excellent figures.

Dr. James Ritchie gives a good account of a remarkable and little understood disease, general cystic degeneration of the kidneys. It is illustrated by many good drawings, and forms the best essay on the subject with which we are acquainted. There is an appendix of 88 collected cases, and a list of the literature. Dr. Ritchie thinks that these kidneys should be classed with the adenomata; that they are closely allied to cystic livers, but are not connected with congenital cystic kidney. He seems to us to bring forward strong evidence that the view he takes of the pathology of this affection is the correct one. The theory that regards this form of cystic disease of the kidney as an extreme development of the cysts so often met with in chronic interstitial nephritis has little, in our opinion, to support it.

If we have thus selected a few of the papers for brief remark, it is not that the remaining ones are not also excellent. We must allude again to the beautiful illustrations, and also give the praise which is due to the clearness of the type and the neat and handsome manner in which the volume is turned out. It is a book which we read with much interest and profit, and a useful feature of it is the bibliography which follows each paper.

